# Kenyan *Orthosiphon schimperi* Benth. Essential Oil: Chemical Composition and Cytotoxic Activity on HeLa Cells

**DOI:** 10.3390/plants14223513

**Published:** 2025-11-18

**Authors:** Natale Badalamenti, Viviana Maresca, Antonella Porrello, Vincenzo Ilardi, Alessia Postiglione, Martina Dentato, Elena De Marino, Alessandra Pollice, Maurizio Bruno

**Affiliations:** 1 Department of Biological, Chemical and Pharmaceutical Sciences and Technologies (STEBICEF), Università degli Studi di Palermo, Viale delle Scienze, Ed. 17, 90128 Palermo, Italy; natale.badalamenti@unipa.it (N.B.); antonella.porrello@unipa.it (A.P.); vincenzo.ilardi@unipa.it (V.I.); 2 National Biodiversity Future Center, (NBFC), 90133 Palermo, Italy; 3 Department of Life Sciences, Health and Health Professions, University of Rome “Link Campus”, 00165 Rome, Italy; 4 Department of Biology, University of Naples Federico II, Complesso Universitario Monte Sant’Angelo, Via Cinthia 4, 80126 Napoli, Italy; alessia.postiglione@unina.it (A.P.); martina.dentato@unina.it (M.D.); elena.demarino@unina.it (E.D.M.); alessandra.pollice@unina.it (A.P.)

**Keywords:** *Orthosiphon schimperi* Benth., eugenol methyl ether, caryophyllene oxide, cytotoxicity assays, HeLa cells, HaCaT cells

## Abstract

The genus *Orthosiphon* Benth., is a relatively small genus of the Lamiaceae family that includes forty-four accepted species distributed mainly in tropical and sub-tropical areas of Asia, Southern Africa, and Madagascar. The species usually occurs in woodland, grassland, or forest margins. Due to their curative properties, species of this genus have been largely utilized in the popular medicine of several countries. Essential oil from fresh post-flowering aerial parts of *Orthosiphon schimperi* Benth. (**OS**), a taxon not previously studied, was collected in Kenyan territory, and it was obtained by hydrodistillation (yield 0.15%); its chemical profile was investigated by GC–MS analysis, using a DB-5ms low-polarity GC column. Oxygenated monoterpenes dominated the **OS** composition, and eugenol methyl ether (79.5%) was, by far, the main constituent of the sample. Oxygenated sesquiterpenes were the second most abundant class (8.8%), mainly constituted by caryophyllene oxide (8.2%). The biological activity of **OS** was assessed by using cytotoxicity assays on HeLa cells (human cervical cancer cell line) and HaCaT cells (non-tumorigenic human keratinocytes). **OS** exhibited selective cytotoxicity toward HeLa cells (IC_50_ = 29.44 µg/mL after 24 h of treatment), while having minimal or no impact on HaCaT cell viability. Western blot analysis indicates that, indeed, the EO induces selective apoptosis in HeLa cells, thereby suggesting a potential anticancer activity exhibited by *O. schimperi* EO.

## 1. Introduction

The genus *Orthosiphon* Benth, belonging to the Labiatae family, comprises forty-four accepted species growing in tropical and subtropical Asia, Southern Africa, and Madagascar, with a disjointed species, *O. americanus* Harley & A. J. Paton, endemic to Colombia. The species usually occurs in woodland, grassland, or forest margins [1]. The word *Orthosiphon* was coined from the Latin words *orthos* (straight) and *siphon* (cylindrical), based on its morphological features and referring to the straight tube-like flowers that are produced by the *Orthosiphon* species [2]. Several *Orthosiphon* species are utilized as medicinal plants that are used in herbalism due to their curative properties. *O. thymiflorus* (Roth) Sleesen is used in India for its cytotoxic, antidiabetic, anti-inflammatory, and anti-hypertensive properties [3,4,5].

In the Tamilnadu region, the leaves of this plant are traditionally used for their health-promoting effects, particularly in supporting circulation, countering oxidative stress, and controlling insect larvae [3,6]. In India, a decoction prepared from the same species, also known by its synonym *O. glabratus* Benth., is commonly used to address digestive issues such as diarrhea and piles, while the leaves are also applied directly to the skin to aid in healing cuts and wounds [7].

*O. pallidus* Royle ex Benth, an herbaceous shrub native to Southeast Asia and India, has long been valued in traditional medicine. It is used to alleviate symptoms of infectious diseases, joint and liver disorders, urinary conditions, and other systemic imbalances [8,9]. Recognized in Ayurvedic practices and referred to by the folk names ajagur and naganda-baavari, this plant is also employed to ease discomfort associated with painful urination and abdominal cramps [10].

In the Randa Region of Djibouti, local communities use crushed leaves of *O. pallidus*, known there as abursaafiqi and ganduwayto, by soaking them in water and drinking the infusion. This remedy is believed to provide relief from sunburns, kidney problems, gastrointestinal discomfort, internal parasites, and even snake bites [11].

By contrast, in areas such as Baluchistan, Arabia, and India, traditional healers prefer using the entire plant. It is typically prepared and administered as a restorative remedy for mental fatigue and is also believed to act as both a general health tonic and an aphrodisiac [12].

In Burkina Faso, the dried seeds of *O. pallidus*, known as *ninkaansega*, are used for cleaning the eyes, whereas the leaves and stems are utilized for breast pain, gout, and to improve the quality of milk of women who have recently delivered [13]. *O. diffusus* (Benth.) [synonym of *Endostemon viscosus* (Roth) M. Ashby] is largely used in the folk medicine of Western Ghats, India, where it is known by the vernacular name *senthulasi*, for treating hepatitis, inflammation, and jaundice [14]. *O. aristatus* (Blume) Miq. has been used for several centuries in the ethnomedicine of Indonesia, Malaysia, and Southeast Asia, for the treatment of diabetes, kidney stones, and hypertension [15,16,17]. Furthermore, *O. aristatus* possesses diuretic properties and is used in treating urinary lithiasis, edema, eruptive fever, influenza, rheumatism, hepatitis, and jaundice [18]. In Mauritius, a decoction of a few leaves of *O. aristatus*, with the vernacular name *autochiffon*, administered in small portions orally throughout the day, is utilized for asthma, bronchitis, and respiratory disorders [19].

*O. stamineus* Benth., syn. *O. aristatus* var. *aristatus* has been traditionally utilized in many South-East Asian countries, such as Thailand, Malaysia, Indonesia, Japan, and Myanmar, to treat several illnesses. In Indonesia, the leaves have been used for treating rheumatism, diabetes mellitus, and hypertension, and as a diuretic and antipyretic for menstrual disorder, gonorrhea, syphilis, acute and chronic nephritis, and gout. Both in Vietnam and Malaysia, the aerial parts are used for treating influenza, hepatitis, jaundice, urinary lithiasis, and biliary lithiasis. Its properties as a diuretic, kidney tonic, and antidiabetic have also been reported in Myanmar and Thailand [20]. The poultice or decoction of the leaves of *O. hildebrandtii* Vatke (*Ekebunga baiseke*) is used in Kenya for stomach and oral cavity infections [21].

Non-volatile organic compounds identified in the extracts of *Orthosiphon* ssp. includes flavonoids, phenyl acids, chromones, lignans, alkaloids, sesquiterpenes, triterpenoids, and many diterpenoids with a variety of skeletons, mainly isopimarane, staminane, secoisopimarane, secostaminane, and norstaminane [22,23,24].

Several pharmacological studies showed that the extracts possess a large number of properties both in vitro and in vivo, including anti-viral, anti-Alzheimer’s disease, anti-inflammatory, antitumor, anti-proliferative, and antiangiogenic, antibacterial activity, antidiabetic, anti-inflammatory, antioxidant, hepatoprotective, analgesic, nephroprotective, and so on [22,23,24,25].

**Figure 1:**
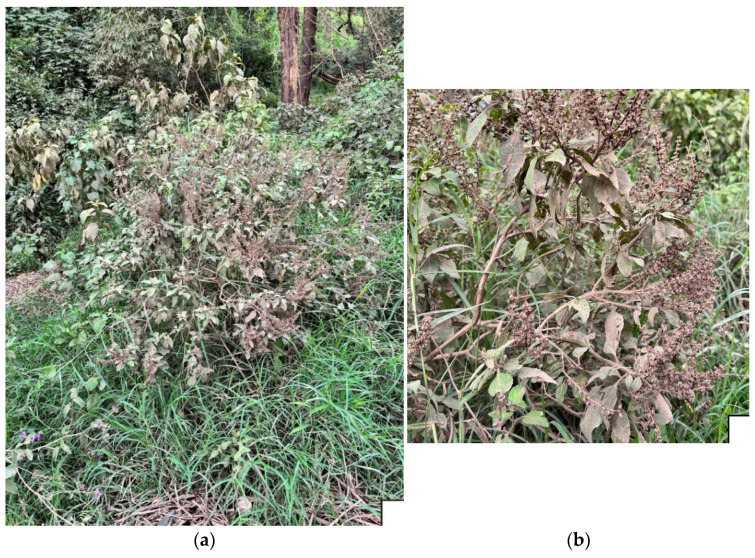
(**a**): *Orthosiphon schimperi* Benth. plants collected in Nakuru Lake Park, Kenya; (**b**): Details of the top aerial parts in the post-inflorescence period. Photos by Prof. Maurizio Bruno.

In this context, this work focuses on the investigation of the properties of *Orthosiphon schimperi* Benth. [syn.: *Ocimum buchananii* Baker; *Ocimum coloratum* Hochst.; *O. buchananii* (Baker) M.R.Ashby, *O. dissimilis* N.E.Br., *O. shirensis* Baker], not previously studied, in order to expand the pharmacological potential knowledge on *Orthosiphon* species. *O. schimperi* Benth. (Figure 1a,b) is a perennial herb that is up to 60 cm in length, with leaves ovate-lanceolate with dentate margins, with inflorescences terminal in most parts purple and with flowers white or mauve; it takes its name, *schimperi*, from Wilhelm Schimper, explorer, collector, and active researcher in the Ethiopian regions. This plant is widespread in Tropical Africa to Limpopo, South Africa, and it grows in grassy areas in open woodland between 450 and 1650 m s.l. [26].

In particular, in this study, the *O. schimperi* essential oil (**OS**) was obtained from fresh post-flowering aerial parts and was both chemically and biologically investigated, highlighting the cytotoxic effects of the whole essential oil and of the major compound, eugenol methyl ether, on HeLa cells (human cervical cancer cell line) and HaCaT cells (non-tumorigenic human keratinocytes).

## 2. Results and Discussion

### 2.1. Gas Chromatography and Mass Spectrometry (GC-MS) Analysis of the Essential Oil

The composition of the EO of *O. schimperi* (**OS**) was analyzed by GC-MS analysis (Table 1). Twenty-one compounds, divided into four classes, were identified and classified according to linear retention indices (LRIs). A total of 96.68% of the total composition was correctly identified by comparison with linear retention indices (LRIs) and mass spectra (as reported in Section 3.4), and the major compounds, eugenol methyl ether and caryophyllene oxide, were confirmed by injection of authentic standards. Only 3.1% of the total compounds were unidentified, but the mass spectra showed patterns typical of oxygenated sesquiterpenes. In terms of compound classes, oxygenated monoterpenes (83.47%) dominate, by far, **OS**, with eugenol methyl ether as the most abundant compound (79.38%). This exceptionality may be due to several environmental and climatic factors. Samples were, in fact, harvested near the end of their lifespan, during the dry season, when temperatures average no higher than 25 °C during the day and 8 °C at night.

Among the oxygenated sesquiterpenes (8.64%), only caryophyllene oxide (8.00%) is present in significant quantity. On the other hand, hydrocarbons, both monoterpenic and sesquiterpenic, occur in very moderate amounts (3.06% and 1.51%, respectively).

As shown in Table 2, all the species so far have an Asian geographical origin, and up to now, no African taxa have been investigated for the chemical composition of their EO. 

**Table 2:** Main components (>3%) of *Orthosiphon* taxa EOs from different origins previously reported in the literature.

Taxa	Parts	Origins	Main Components (%)	Compoud Classes	Ref.
*O. aristatus* var. *aristatus*	lv	Commercial	Palmitic acid (9.51%) *β*-Selinene (7.84%) *β*-Caryophyllene (6.45%) *δ*-Cadinene (6.33%) Caryophyllene oxide (6.05%) Hexahydrofarnesyl acetone (5.76%) *β*-Elemene (4.92%) *α*-Humulene (3.37%)	O SH SH SH OS O SH SH	[27]
*O. diffusus* (Benth) Benth. *	lv	India	*n*-Eicosane (19.5%) *β*-Caryophyllene (18.6%) *n*-Octocosane (12.2%) Limonene (11.6%) *β*-Ocimene (4.2)	O SH O MH MH	[28]
*O. pallidus* Royle, ex Benth	ap	India	*β*-Caryophyllene (17.4%) 7-*epi-α*-Selinene (15.2%) Terpinolene (6.9%) *β*-Pinene (6.8%) *β*-Elemene (5.1%) *α*-Humulene (4.9%) *α*-Copaene (4.8%) *epi*-Cubebol (4.5%) Zonarene (3.9%)	SH SH MH MH SH SH SH OS SH	[29]
*O. stamineus* Benth. **	ap	Malaysia	*β*-Caryophyllene (26.31%) Bicyclogermacrene (7.7%) Eugenol methyl ether (7.4%) *α*-Humulene (5.06%) *α*-Copaene (2.26%)	SH SH OM SH SH	[30]
	lv	Malaysia	*β*-Caryophyllene (24.0%) *α*-Humulene (14.2%) *β*-Elemene (11.1%) 1-Octen-3-ol (8.2%) *β*-Bourbonene (3.4%)	SH SH SH O SH	[31]
	st	Malaysia	*β*-Caryophyllene (35.1%) *α*-Humulene (18.4%) *β*-Elemene (8.5%) 1-Octen-3-ol (7.0%) *β*-Bourbonene (3.0%)	SH SH SH O SH	[3]
*O. thymiflorus* (Roth) Sleesen	lv	India	2-Isopropyl-5-methyl-9-methylene-bicyclo-1-decene (4.4.0) (42.62%) Carotol (16.48%) *α*-Cadinol (6.14%) *δ*-3-Carene (5.15%) *α*-Humulene (3.61%) *γ*-Cadinene (3.34%)	SH OS OS MH SH SH	[32]
	ap, r	India	Isobornyl acetate (55.36%) Nerolidol (7.19%) Camphene (5.52%) Eugenol (4.15%) *α*-Pinene (4.07%)	O OS SH OM MH	[33]

ap = aerial parts; lv = leaves; st = stems; r = roots; * synonymous with *Endostemon viscosus* (Roth) M.R.Ashby; ** syn. with *Orthosiphon aristatus* var. *aristatus;* MH: Monoterpene hydrocarbons; OM: Oxygenated monoterpenes; SH: Sesquiterpene hydrocarbons; OS: Oxygenated sesquiterpenes; O: Other compounds.

The composition of **OS** totally differs from the other previously studied ones. In fact, the main constituent of **OS**, eugenol methyl ether, has been identified only, and in quite a small amount (0.2–0.3%), in the EOs from leaves and stems of *O. stamineus* Benth. (syn. *O. aristatus* var. *aristatus*), collected in Malaysia [31] , and in the EO of another accession from Malaysia of the same species (7.4%) [30]. 

The EO of *O. pallidus* Royle, ex Benth., collected in India, was found to be very rich in sesquiterpene hydrocarbons with *β*-caryophyllene (17.4%) as the main constituent [29]. This compound was also reported among the principal metabolites of EOs of two accessions of *O. stamineus* from Malaysia (24.0–35.1%) [30,31] and of *O. diffusus* (Benth) Benth. [syn. *Endostemon viscosus* (Roth) M. R. Ashby] (18.6%) [28], but totally absent in **OS**. Caryophyllene oxide, the second most abundant compound in **OS**, has been identified only in the EO from the commercial plant of *O. aristatus* var. *aristatus* [27].

### 2.2. Cytotoxicity of OS

**Figure 2:**
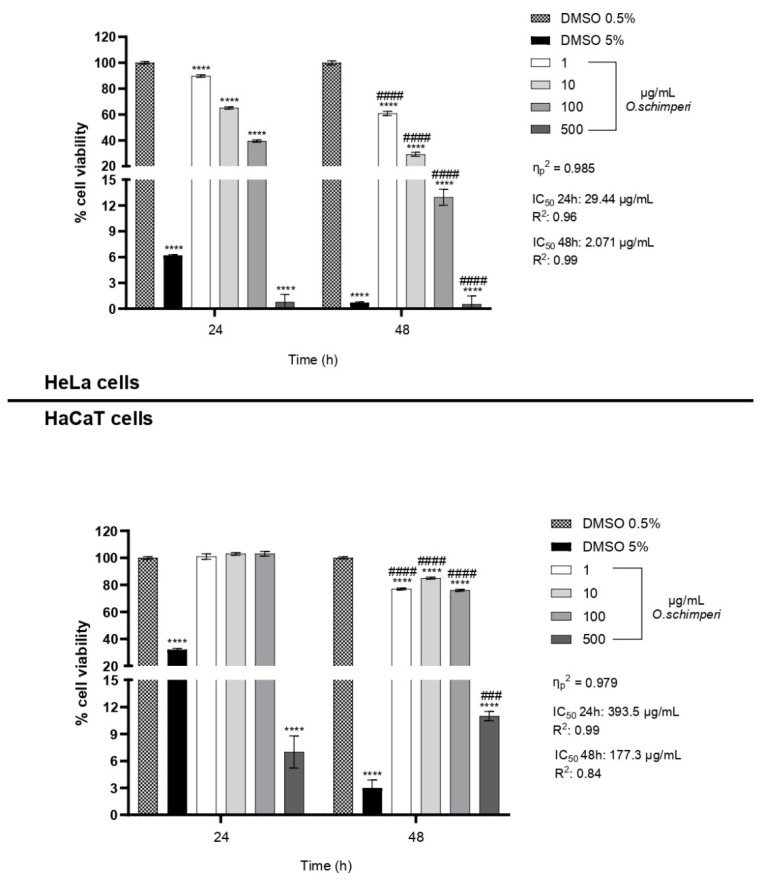
Effect of **OS** on cell viability of HeLa and HaCaT cells at different concentrations (0, 1, 10, 100, and 500 µg/mL) after 24 and 48 h of treatment. For HeLa cells, IC_50_ values were 29.44 µg/mL (24 h, R^2^ = 0.96) and 2.071 µg/mL (48 h, R^2^ = 0.99). For HaCaT cells, IC_50_ values were 393.5 µg/mL (24 h, R^2^ = 0.99) and 177.3 µg/mL (48 h, R^2^ = 0.84), as shown in Figure S1. Effect size was reported as η^2^p value. Data were presented as mean ± SD (*n* = 3). Each sample was analyzed in three technical replicates. Statistical significance was determined by two-way ANOVA: * indicates statistical significance compared to the corresponding control (DMSO 0.5%) at the same exposure time (24 h or 48 h); # indicates statistical significance between 24 h and 48 h within the same concentration. **** *p*-value < 0.0001, ### *p*-value < 0.001, and #### *p*-value < 0.0001.

**OS**, as shown in Figure 2, demonstrated remarkable and consistent cytotoxic activity when applied to human cervical cancer cells (HeLa), while simultaneously exhibiting only minimal levels of toxicity toward non-tumorigenic human keratinocytes (HaCaT). This differential cytotoxic response observed between the two cell lines strongly suggests a mechanism of selective cytotoxicity, which is widely recognized as a critical property for any candidate compound intended for anticancer therapy. The ability of a compound to specifically target and eliminate malignancy, while preserving the viability and integrity of healthy, non-cancerous cells, is a fundamental prerequisite for reducing the adverse side effects typically associated with conventional chemotherapeutic agents and improving the therapeutic index of novel interventions.

The results obtained from the cytotoxicity assays revealed a clear and reproducible pattern of dose- and time-dependent reduction in HeLa cell viability following exposure to **OS**. After 24 h of treatment, a gradual but measurable decline in cancer cell viability was observed across the concentration range of 1 to 100 µg/mL. However, at the highest tested concentration of 500 µg/mL, a dramatic and almost complete loss of viability occurred, with residual cell survival falling below 2%. This effect became even more pronounced and severe after 48 h, where a marked decrease in cell viability was evident even at lower concentrations, with viability dropping below 15% at just 100 µg/mL. These results point to a cumulative or sustained effect of **OS** over time, suggesting that prolonged exposure enhances the biological activity of the oil, possibly through progressive intracellular accumulation or amplification of cytotoxic signaling pathways.

Conversely, the response of HaCaT cells to **OS** was strikingly different. After 24 h of exposure, HaCaT cells maintained greater than 80% viability at all concentrations tested except 500 µg/mL; after 48 h of treatment, cell viability remained greater than 70% except at the highest dose of 500 µg/mL. This observation indicates a robust resistance of normal keratinocytes to the cytotoxic effects of **OS**, further supporting the idea that the essential oil exerts targeted activity predominantly against transformed or cancerous cells. These findings are in agreement with several studies in the literature reporting that essential oils derived from aromatic and medicinal plants frequently demonstrate selective toxicity toward cancer cells, often mediated through the induction of oxidative stress, DNA fragmentation, and apoptotic cell death mechanisms [33,34,35].

**Figure 3:**
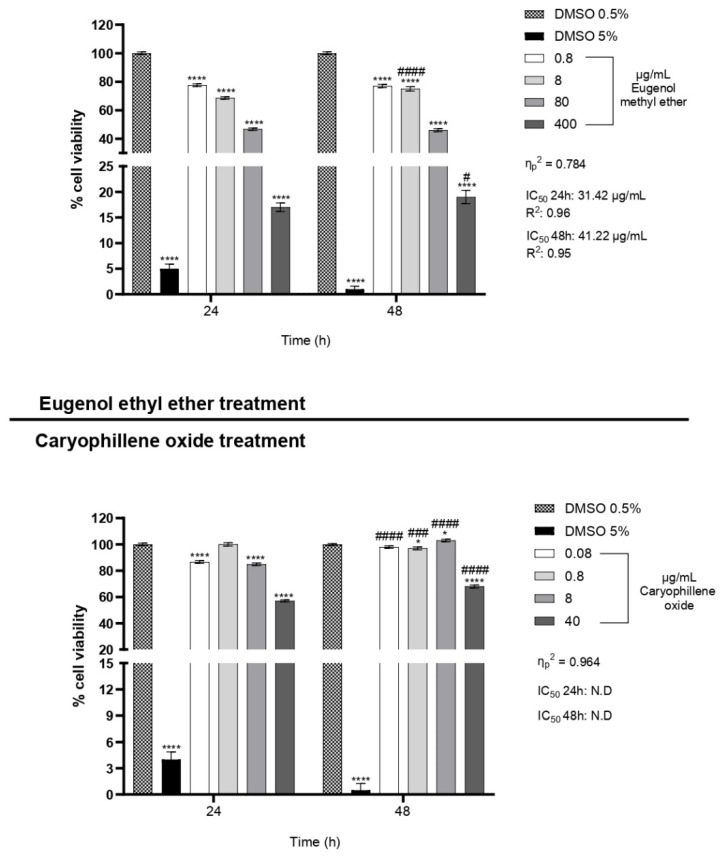
Cytotoxic effects of eugenol methyl ether and caryophyllene oxide on HeLa cells after 24 and 48 h of treatment. HeLa cells were treated with increasing concentrations of eugenol methyl ether (0, 0.8, 8, 80, and 400 µg/mL), and the IC_50_ values were 31.42 µg/mL (24 h, R^2^ = 0.96) and 41.22 µg/mL (48 h, R^2^ = 0.95), as shown in Figure S2, and caryophyllene oxide (0, 0.08, 0.8, 8, and 40 µg/mL). Data were expressed as mean ± SD (*n* = 3). Each sample was analyzed in three technical replicates. * indicates statistical significance compared to the corresponding control (DMSO 0.5%) at the same exposure time (24 h or 48 h). # indicates statistical significance between 24 h and 48 h within the same concentration. * *p*-value < 0.05, **** *p*-value < 0.0001, # *p*-value < 0.05, ### *p*-value < 0.001, and #### *p*-value < 0.0001.

To identify the specific constituents responsible for the observed bioactivity of **OS**, the two major components—eugenol methyl ether and caryophyllene oxide—were individually tested on HeLa cells. Each compound was diluted proportionally to its relative abundance in the EO and evaluated in cytotoxicity assays. Eugenol methyl ether, which represents the predominant compound in **OS**, exhibited a strong and dose-dependent reduction in HeLa cell viability. After just 24 h of exposure, a significant drop in viability occurred at concentrations as low as 0.8 µg/mL. At 400 µg/mL, cell viability dropped below 20%, and the same trend was observed after 48 h of treatment. This time-enhanced effect, observed even at lower concentrations, suggests that eugenol methyl ether exerts a sustained cytotoxic impact, potentially due to its ability to penetrate cell membranes efficiently and initiate apoptosis through the accumulation of intracellular reactive species (Figure 3).

The anticancer potential of eugenol and several of its derivatives has been well documented in previous research. These compounds are known to act via multiple mechanisms, including mitochondrial membrane depolarization, excessive generation of reactive oxygen species (ROS), and the activation of caspase-dependent apoptotic pathways [36]. The high lipophilicity of eugenol derivatives also facilitates their rapid and effective diffusion across lipid bilayers, allowing them to accumulate in target organelles and exert pronounced bioactivity [37].

In contrast, caryophyllene oxide showed none or only moderate cytotoxic activity against HeLa cells under the same conditions. A limited reduction in viability was observed only at the highest tested concentration (40 µg/mL), with no significant effect at lower doses. Although caryophyllene oxide has previously been reported to induce apoptosis through endoplasmic reticulum stress and ROS-mediated signaling cascades [38], its significantly lower abundance in the EO and reduced potency in this context suggest that it likely plays a supportive or secondary role in the overall cytotoxic profile of **OS**.

The higher bioactivity of the whole EO compared to its isolated constituents can be attributed to synergistic interactions among its multiple volatile compounds. Harris et al. [39] introduced the concept of aromatic synergy, proposing that EO components can act cooperatively, producing effects greater than the sum of their individual actions.

Experimental evidence supports this notion. Khodaei et al. [40] demonstrated that the chemical diversity of EOs, including both major and minor constituents, enhances biological effects through interactions that potentiate activity. Similarly, Bassolé and Juliani [41] summarized antimicrobial studies showing that combinations of EOs or their components often outperform individual compounds, highlighting the importance of mixture effects in biological activity.

Mechanistic studies further illustrate these interactions. Połeć et al. [42] investigated specific terpenes from hop EO (*β*-myrcene, α-humulene, *β*-farnesene, and limonene) and found that their interactions can be synergistic, additive, or antagonistic, depending on ratios and concentrations, emphasizing that the composition context is critical for bioactivity.

EOs are thus best conceptualized as multicomponent systems, whose emergent properties—such as improved stability, enhanced permeability, and action on multiple targets—cannot be predicted by summing the effects of isolated compounds [43]. Brandes et al. [44] also showed that individual volatile compounds often do not match the antimicrobial potency of whole oil mixtures, demonstrating that synergy among constituents is a biologically relevant and measurable phenomenon.

Overall, these observations support our results: the superior activity of the complete EO likely arises from the cooperative and context-dependent interactions among major and minor constituents, producing a more potent and multifaceted biological effect than any single component alone.

### 2.3. **OS** Induces PARP-1 Cleavage in HeLa Cells

**Figure 4:**
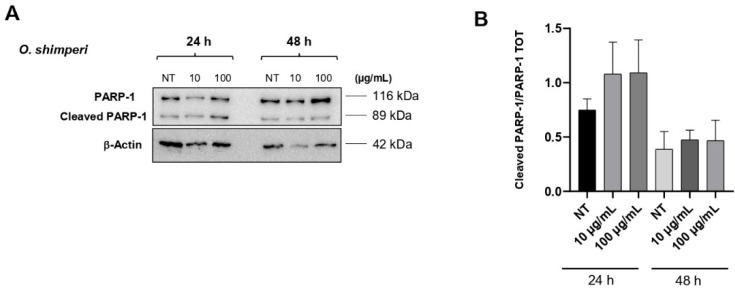
Effect of **OS** on PARP-1 cleavage in HeLa cells at 24 and 48 h of treatment. (**A**): HeLa cells were incubated with or without **OS** at 10 and 100 µg/mL for 24 or 48 h. Cells were analyzed by Western blot with antibodies against cleaved PARP-1 and *β*-actin; (**B**): Quantification of normalized cleaved PARP-1 levels from three independent replicates performed by ImageJ BioRad software.

To gain further insight into the mechanisms by which **OS** induces cytotoxicity, it was investigated whether cells undergo apoptosis upon exposure to the complete **OS**, since it exhibits a greater cytotoxic effect than the isolated components. The induction of the apoptotic program was assessed by analyzing the proteolytic cleavage of PARP-1 (Poly ADP-ribose polymerase 1) after exposure of cells to two different concentrations of **OS** (10 and 100 μg/mL), which were selected for their ability to exert a biological effect without inducing massive cell death. As shown in Figure 4A,B, the proteolytic cleavage of PARP-1 (which is also detectable in untreated cells, see lane 1) was markedly increased by treatment with **OS** at 24 h, while it became less evident at 48 h, as PARP-1 cleavage is a relatively early event in the apoptotic process. In agreement with the absence of toxicity in HaCat cells (see Figure 2), no induction of the apoptotic program in these cells was observed. Furthermore, the lack of p21^Cip1/Waf1 induction under the same conditions provides additional confirmation that the cells did not enter cell cycle arrest, but rather likely underwent cell death. Although upstream apoptosis markers such as caspase-9 and caspase-3 have not yet been analyzed, PARP-1 cleavage serves as a robust indicator of the cell’s commitment to apoptosis, thereby highlighting the significance of our observations and suggesting the cellular mechanism through which **OS** exerts its function in HeLa cells. The observation that the complete **OS** exhibits greater cytotoxic efficacy than either of its isolated components strongly supports the hypothesis that synergistic or additive interactions among various phytochemicals may contribute to its biological effects. EOs are complex mixtures that often rely on the interplay between major and minor constituents to exert full therapeutic potential. These interactions can enhance membrane fluidity, stabilize radical species, or modulate intracellular signaling cascades in ways that exceed the capability of individual compounds that act alone [45]. Similar synergistic effects have been described in other members of the Lamiaceae family, where whole-plant extracts often outperform purified constituents in both in vitro and in vivo models of disease [46]. This reinforces the concept that whole extracts may offer broader and more effective therapeutic outcomes than isolated molecules, particularly in the context of complex diseases such as cancer.

## 3. Materials and Methods

### 3.1. Plant Materials

The post-flowering aerial parts of *Orthosiphon schimperi* were collected in Nakuru Lake Park, Makalia Falls View Point, Kenya (0°29′30″ S; 36°04′51″ E, 1830 m s/l.), on 13 August 2024. In an area of approximately 10 m^2^, six specimens were collected and used to obtain the **OS**. Environmental conditions: dry season, and during this time the skies are clear, rainfall is minimal, and temperatures are mild; weather conditions: daytime temperatures typically range between 20 °C and 25 °C (68 °F to 77 °F), while nights can be cooler, dropping to 10 °C to 12 °C (50 °F to 54 °F).

One of the samples, identified by Prof. Vincenzo Ilardi, was stored in the Herbarium Mediterraneum Panormitanum (PAL) (Voucher N.109966) of the Botanical Garden of the University of Palermo, Italy.

### 3.2. Isolation of EO

Fresh post-flowering aerial parts (250 g) were subjected to hydrodistillation with distilled water (800 g) for 3 h, according to the standard procedure described in European Pharmacopeia [47]. Samples yielded 0.15% of EO.

### 3.3. Chemicals

The synthesis of eugenol methyl ether was carried out, starting from commercial eugenol. A total of 30 mg of eugenol was dissolved in dry tetrahydrofuran, and it reacted with diazomethane in large amounts, under stirring conditions at room temperature for 24 h. Diazomethane, easy to use and rapidly reactive, is one of the best methylating agents because, unlike other methylating agents, it only replaces strongly acidic protons, as in this case. Reaction progress was monitored by thin-layer chromatography on silica gel plates (Merck 60, F254, 0.2 mm) until the total conversion of eugenol in eugenol methyl ether. The purity of the synthesized compound was verified by GC-MS analysis.

All mentioned chemicals, including caryophyllene oxide, were purchased from Sigma-Aldrich Srl (Milan, Italy).

### 3.4. GC-MS Analysis

GC-MS analysis of the EO was carried out using a Shimadzu QP 2010 plus equipped with an AOC-20i autoinjector (Shimadzu, Kyoto, Japan) gas chromatograph equipped with a capillary non-polar column (DB-5-MS) 30 m × 0.25 mm i.d., film thickness 0.25 μm and a data processor, working with the following temperature program: 5 min at 40 °C, and subsequently at 2 °C/min up to 260 °C, held for 20 min; injector and detector temperatures, 280 °C; carrier gas, helium (1 mL/min); injection volume of 1 µL, split ratio, 1:50; scan time: 175 min; acquisition mass range: 29–400 amu. The percentage values of volatile components were the average of three injections of each **OS** sample. A total of three different batches of **OS** were used.

All mass spectra were acquired in electron-impact (EI) mode with an ionization voltage of 70 eV. The pressure was 35 kPa at constant pressure. The carrier gas was helium, and u was 32.12 cm/s. The settings were as follows: ionization voltage, 70 eV; electron multiplier energy, 2000 V; transfer line temperature, 295 °C; and a solvent delay of 3 min.

The identification of volatile components was based on computer matching with the WILEY275, NIST05, and ADAMS libraries, as well as by comparison of the mass spectra and linear retention indices (LRIs) with those reported in the literature (https://webbook.nist.gov/ (accessed on 10 September 2025)). In particular, for the identification, two different filters were used: the first one was based on minimum MS spectral similarity (≥89%); the second filter, instead, concerned the comparison between experimental and theoretical LRI values. Each result was accepted only if it presented a tolerance window of ±10 units between the experimental and theoretical LRI values; even in this case, a satisfactory ΔLRI was obtained for all the identified components, with an average ΔLRI of 2.4.

Additionally, eugenol methyl ether and caryophyllene oxide, majority compounds, were confirmed by injection of authentic standards in our possession and by mass comparison.

### 3.5. Cell Viability Assay (CCK-8)

The cytotoxic activity of **OS** was evaluated using the Cell Counting Kit-8 (CCK-8; Dojindo Molecular Technologies, Kumamoto, Japan), following the manufacturer’s instructions.

HeLa cells (human cervical cancer) were purchased from ATCC (CCL-2), while HaCaT (immortalized human keratinocyte) cells were purchased from CLS Cell Lines Service Germany. Both cell lines were seeded in 96-well plates at a density of 3 × 10^3^ cells/well in 100 μL of complete Dulbecco’s Modified Eagle Medium (DMEM) and incubated at 37 °C in a 5% CO_2_ humidified atmosphere for 24 h to allow cell adherence.

Cells were then treated with increasing concentrations of **OS** (1, 10, 100, and 500 µg/mL, diluted in DMSO with the final DMSO concentration 0.5%) and incubated for 24 and 48 h. Untreated cells (DMEM + 0.5% DMSO) were used as negative controls. Wells containing medium and CCK-8 reagent without cells were included as blanks.

After treatment, 10 μL of CCK-8 reagent was added to each well, and the plates were incubated for 2 h at 37 °C. The absorbance was measured at 450 nm using a microplate reader (Bio-Rad, Thermo Fisher).

In addition to **OS**, its two main constituents—eugenol methyl ether and caryophyllene oxide—were individually tested on HeLa cells under the same experimental conditions. The selected concentrations for these compounds (80% for eugenol methyl ether and 8% for caryophyllene oxide) were chosen to reflect their proportional abundance in the complete essential oil, as determined by GC-MS analysis.

For all treatments, dose–response curves were generated, and the half-maximal inhibitory concentration (IC_50_) values were calculated using nonlinear regression analysis with GraphPad Prism (version 10). Each IC_50_ measurement was performed in triplicate to ensure reproducibility and reliability of the data. The IC_50_ values were calculated using GraphPad Prism software, applying the standard nonlinear regression formula:

$$y=\frac{100}{1+{\left(\frac{{IC}_{\left\{50\right\}}}{x}\right)}^{\left\{HillSlope\right\}}}$$

where y represents the percentage of cell viability, x is the concentration of the compound, and HillSlope describes the steepness of the dose–response curve.

### 3.6. Western Blot Analysis

HeLa cells and HaCaT cells were harvested in RIPA buffer and 1X Protease Inhibitor Cocktail (Sigma-Aldrich, St. Louis, MO, USA) and processed as described [48]. Western blot analyses were performed following standard procedures for apoptotic marker detection, including PARP-1, with slight modifications of the protocol described by Chianese et al. [49]. Proteins were then transferred to a Protran^®^ Nitrocellulose membrane (Sigma-Aldrich, St. Louis, MO, USA) using a Mini trans-blot apparatus (Bio-Rad, Hercules, CA, USA) according to the manufacturer’s instructions. The membrane was then incubated with the indicated antibodies. Primary antibodies were anti-rabbit cleaved PARP-1 (Cell signaling EuroClone, Milan, Italy 95415-S) and anti-mouse *β*-actin (C4 Santa-Cruz Biotechnology, Dallas, TX, USA SC-47778). Secondary antibodies were anti-rabbit HRP (Merck Millipore, Billerica, MA, USA 12-348) and anti-mouse (Merck Millipore, Billerica, MA, USA 12-349). Proteins were visualized by enhanced chemiluminescence (ECL, Bio-Rad, Hercules, CA, USA) and revealed by Quantity One software of ChemiDoc TM XRS system (Bio-Rad, Hercules, CA, USA). Band intensities were quantified by ImageJ BioRad software (Version 2.9.0), normalized with respect to loading controls, and reported as fold increase/reduction with respect to the control sample. Representative experiments are shown for each blot.

### 3.7. Statistical Analysis

Data were first evaluated for normality using the Shapiro–Wilk test and for homogeneity of variances. When assumptions were met, a two-way ANOVA (dose × time) was applied to assess treatment effects. Post hoc comparisons were conducted using Tukey’s multiple comparisons test. Effect sizes were calculated as partial eta squared (η^2^p), using the ratio of the sum of squares for the interaction term divided by the sum of squares for the interaction plus the residuals, according to the following formula:

$$\eta_{p}^{2}=\frac{{SS}_{interaction}}{{SS}_{residual\;}+\;{SS}_{inter\underline{a}ction\;}}$$

Effect size values are reported in Figure 2 and Figure 3.

In all Figures, values are presented as mean ± SD. * indicates statistical significance compared to the corresponding control at the same exposure time. # indicates statistical significance between different times within the same concentration. * *p*-value < 0.05, **** *p*-value < 0.0001, # *p*-value < 0.05, ### *p*-value < 0.001 ####, and *p*-value < 0.0001.

Data were analyzed using the software GraphPad Prism version 10 (San Diego, CA, USA).

## 4. Conclusions

The present study focuses on determining the yield, chemical composition, and biological activity of the essential oil (EO) derived from *O. schimperi*, a species not previously characterized for its phytochemical or pharmacological properties. The findings reveal that the EO possesses notable cytotoxic properties, particularly against human cervical cancer cells (HeLa), while exerting minimal detrimental effects on non-tumorigenic human keratinocytes (HaCaT cell line). This selective cytotoxicity is of considerable therapeutic interest, as it indicates potential for targeting malignant cells without harming normal tissues.

The observed biological activity is primarily attributed to the remarkably high content of eugenol methyl ether, which accounts for nearly 80% of the EO composition. This compound exhibited pronounced dose- and time-dependent cytotoxicity, consistent with previous reports of its ability to induce apoptosis and oxidative stress in cancer cells. These mechanisms may involve mitochondrial dysfunction, reactive oxygen species (ROS) generation, and modulation of key apoptotic regulators. In addition, caryophyllene oxide, another component of the EO, contributed moderately to the overall cytotoxic effect, although its impact was less pronounced. The increased efficacy of the entire EO compared to its individual components suggests that synergistic or additive interactions among the various constituents may enhance the therapeutic potential of the oil as a whole.

Taken together, the results of this study position the essential oil of *O. schimperi*, particularly due to its dominant eugenol methyl ether content, as a strong candidate for further development as a plant-based anticancer agent. Its ability to selectively target cancer cells, combined with its consistent potency over time, underscores its value in natural product-based drug discovery. However, before any clinical application can be considered, additional research is necessary. Future investigations should include detailed studies on the molecular mechanisms underlying its cytotoxicity, comprehensive in vivo assessments to evaluate efficacy and systemic toxicity, and comparative analyses across a broader range of cancer cell lines to better define its spectrum of action and tissue selectivity.

Moreover, exploring the possible synergistic interactions among EO constituents could provide valuable insight into optimizing its anticancer effects. Altogether, these findings contribute to the growing body of evidence supporting the use of medicinal plant-derived essential oils in cancer research and open new perspectives for the utilization of *O. schimperi* as a source of bioactive compounds with potential therapeutic applications.

## Figures and Tables

**Table 1:** Chemical composition of *Orthosiphon schimperi* essential oil (**OS**) collected in Kenya.

No.	Classes and Compounds ^a^	LRI ^b^	LRI ^c^	Area (%)
	**∑ Monoterpene Hydrocarbons (MH)**			**3.06 ± 0.09**
**1**	*α*-Pinene	929	931	0.39 ± 0.01
**2**	*β*-Pinene	970	975	1.57 ± 0.06
**3**	*β*-Myrcene	989	990	0.11 ± 0.00
**4**	*α*-Terpinene	1012	1017	0.13 ± 0.00
**5**	*p*-Cymene	1019	1018	0.11 ± 0.00
**6**	Limonene	1024	1030	0.32 ± 0.01
**7**	*cis*-*β*-Ocimene	1037	1035	0.27 ± 0.01
**8**	*trans*-*β*-Ocimene	1047	1046	0.09 ± 0.00
**9**	*γ*-Terpinene	1055	1053	0.07 ± 0.00
	**∑ Oxygenated Monoterpenes (OM)**			**83.47 ± 3.71**
**10**	Linalool	1099	1097	2.74 ± 0.11
**11**	Isoborneol	1172	1160	0.17 ± 0.00
**12**	*α*-Terpineol	1185	1190	0.42 ± 0.01
**13**	Estragole	1193	1195	0.21 ± 0.01
**14**	Eugenol methyl ether	1413	1401	79.38 ± 3.56
**15**	Isoeugenol methyl ether	1495	1492	0.55 ± 0.02
	**∑ Sesquiterpene Hydrocarbons (SH)**			**1.51 ± 0.04**
**16**	*α*-Cubebene	1345	1346	0.10 ± 0.00
**17**	*α*-Copaene	1371	1373	0.63 ± 0.02
**18**	*α*-Bourbonene	1379	1376	0.36 ± 0.01
**19**	*α*-Bergamotene	1439	1435	0.42 ± 0.01
	**∑ Oxygenated Sesquiterpenes (OS)**			**8.64 ± 0.38**
**20**	Caryophyllene oxide	1578	1578	8.00 ± 0.36
**21**	Alloaromadendrene oxide	1630	1631	0.64 ± 0.02
	**Total**			**96.68 ± 4.22**

^a^ Components listed in the various chemical classes in order of elution on a DB-5-MS non-polar column; ^b^ Linear retention index on a DB-5-MS non-polar column; ^c^ Linear retention indices based on literature (https://webbook.nist.gov/).

## Data Availability

The data presented in this study are available on request from the corresponding author.

## Supplementary Materials

The following supporting information can be downloaded at https://www.mdpi.com/article/10.3390/plants14223513/s1; Figure S1. Dose–response curves of **OS** on HeLa and HaCaT cells. Cell viability was measured after 24 and 48 h of exposure to different concentrations of the **OS** (0–500 µg/mL). For HeLa cells, IC_50_ values were 29.44 µg/mL (24 h, R^2^ = 0.96) and 2.071 µg/mL (48 h, R^2^ = 0.99). For HaCaT cells, IC_50_ values were 393.5 µg/mL (24 h, R^2^ = 0.99) and 177.3 µg/mL (48 h, R^2^ = 0.84). Results are expressed as percentage of cell viability; Figure S2. Dose–response curve of eugenol methyl ether on HeLa cells. Cell viability was assessed after 24 and 48 h of treatment with increasing concentrations of Eugenol methyl ether (0–400 µg/mL). The IC_50_ values were 31.42 µg/mL (24 h, R^2^ = 0.96) and 41.22 µg/mL (48 h, R^2^ = 0.95). Results are expressed as percentage of cells viability.
