# Supplementary material for: Kenyan Orthosiphon schimperi Benth. Essential Oil: Chemical Composition and Cytotoxic Activity on HeLa Cells
Source: Plants (Basel). 2025 Nov 18;14(22):3513. doi: 10.3390/plants14223513 (PMC12656226; doi:10.3390/plants14223513)
Supplement: Supplementary file 1 [file plants-14-03513-s001.zip › plants-3956366-supplementary.pdf]

Supplementary Materials

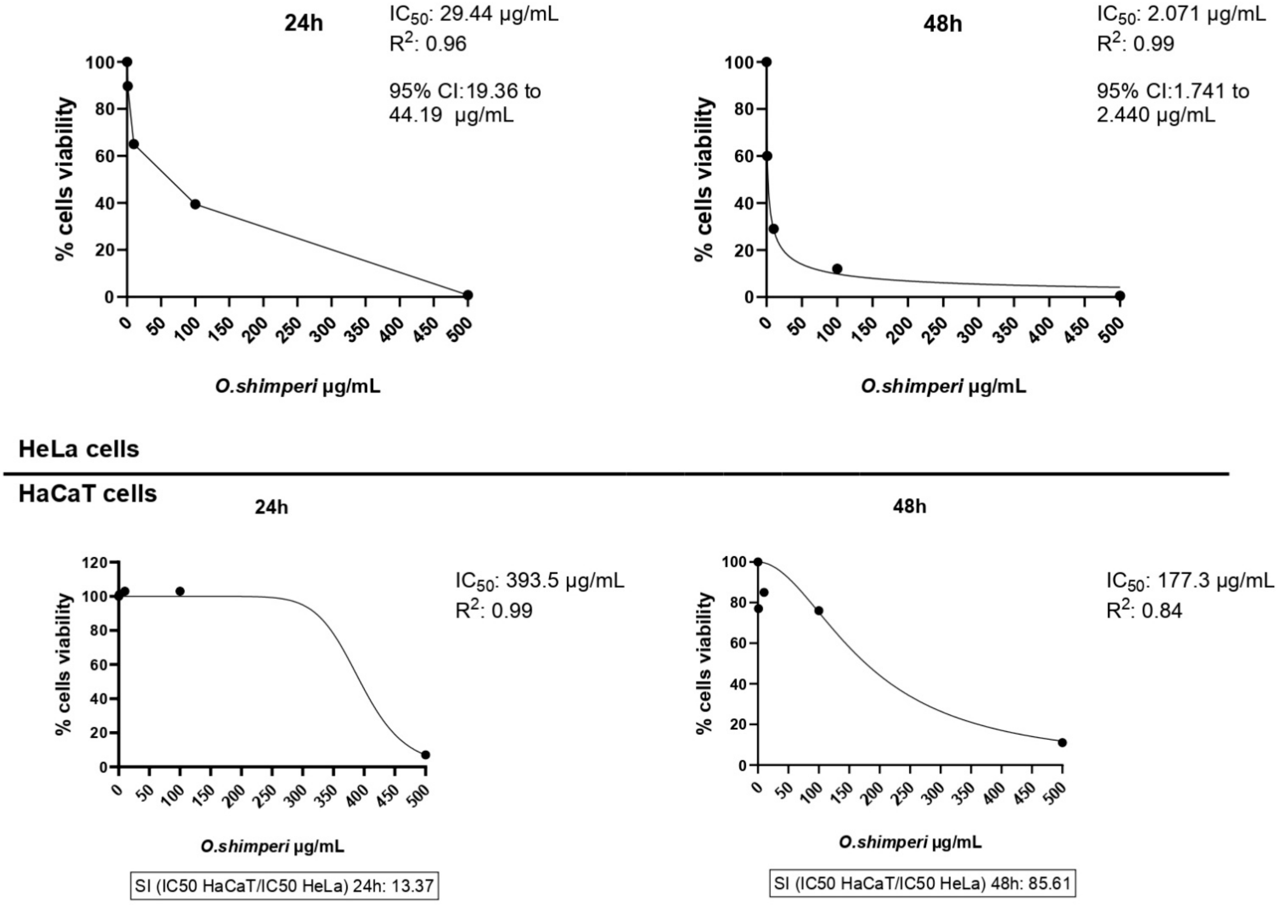

Figure S1. Dose–response curves of **OS** on HeLa and HaCaT cells. Cell viability was measured after 24 and 48 hours of exposure to different concentrations of the oil (0–500 µg/mL). For HeLa cells, IC_{50} values were 29.44 µg/mL (24 h, R^2 = 0.96) and 2.071 µg/mL (48 h, R^2 = 0.99). For HaCaT cells, IC_{50} values were 393.5 µg/mL (24 h, R^2 = 0.99) and 177.3 µg/mL (48 h, R^2 = 0.84). Where applicable, IC_{50} values are shown also with their 95% confidence intervals. Selectivity Index (SI) wcalculated as IC_{50} HaCaT/IC_{50} HeLa at 24h and 48h. Results are expressed as percentage of cells viability.

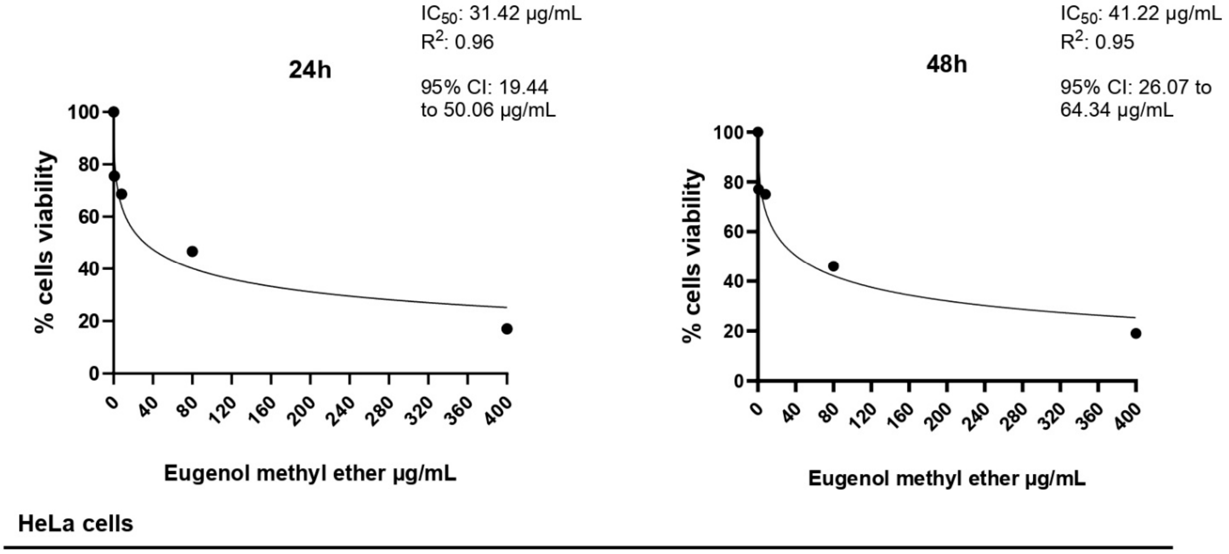

Figure S2. Dose–response curve of eugenol methyl ether on HeLa cells. Cell viability was assessed after 24 and 48 hours of treatment with increasing concentrations of eugenol methyl ether (0–400 µg/mL). The IC_{50} values were 31.42 µg/mL (24 h, R^2 = 0.96) and 41.22 µg/mL (48 h, R^2 = 0.95). Where applicable, IC_{50} values are shown also with their 95% confidence intervals. Results are expressed as percentage of cells viability.
